# Compensatory versus non-compensatory types in myopic acute acquired comitant esotropia: a new classification based on fusion ability at the far point

**DOI:** 10.3389/fmed.2026.1753378

**Published:** 2026-02-23

**Authors:** Yipao Li, Binjun Zhang, Luyao Tong, Na Liao, Huanyun Yu, Fang Zhang, Minghui Wan

**Affiliations:** 1 National Clinical Research Center for Ocular Diseases, Eye Hospital, Wenzhou Medical University, Wenzhou, Zhejiang, China; 2 Department of Ophthalmology, The First Affiliated Hospital of Ningbo University, Ningbo, Zhejiang, China

**Keywords:** acute acquired comitant esotropia, binocular vision, classification, convergence, myopia

## Abstract

**Purpose:**

To propose a novel classification of acute acquired comitant esotropia (AACE) with myopia based on fusion compensation ability at the far point (FP) without myopia correction (MC) and to investigate its pathogenesis in relation to eye-use habits.

**Design:**

A retrospective case-control study.

**Methods:**

This study enrolled 105 myopic AACE patients and 107 control subjects with simple myopia. Data collected included refractive error, angle of deviation (measured by prism and alternate cover test (PACT) and Maddox prism test (MPT) at distance and near), and detailed eye-use habits. AACE patients were categorized into compensatory (*n* = 92) or non-compensatory (*n* = 13) groups based on their ability to maintain binocular fusion at the FP without MC.

**Results:**

A total of 98.10% of AACE patients neglected MC during prolonged near gaze before onset, a rate significantly higher than controls (13.08%). The cohort was divided into compensatory and non-compensatory groups. The compensatory group (87.62% of patients) exhibited distinct features: larger distance versus near deviation (*P* < 0.001), greater MPT than PACT at distance (*P* < 0.001), and near-zero deviation at the FP without MC. In this group, the convergence requirement at the FP (CRFP) showed no significant difference from MPT at distance (MPTD) (*P* = 0.054) and was positively correlated with both MPTD and PACT at distance (PACTD) (*P* < 0.05). In contrast, the non-compensatory group (12.38%) had a younger onset age, lower refractive error, larger deviation angles, and worse stereopsis.

**Conclusion:**

Prolonged near gaze without MC is a critical risk factor for myopic AACE. Classifying patients based on fusion compensation at the FP reveals two distinct subtypes with different clinical profiles. The compensatory type likely represents an abnormal adaptation to excessive convergence demand, whereas the non-compensatory type may indicate a decompensated mechanism. This new classification provides valuable insights for etiology and management.

## Introduction

Acute acquired comitant esotropia (AACE) is characterized by sudden-onset, comitant esotropia, often with diplopia and preserved binocular potential (1–4). The classic Burian and Miller classification delineates three types: Type I (Swan) from fusion disruption, Type II (Burian-Franceschetti) associated with psychosomatic stress, and Type III (Bielschowsky) occurring in uncorrected myopic individuals (1). However, the rising incidence of AACE (5–9), particularly among myopic adults engaged in extensive near work (10, 11), challenges this traditional schema. Etiologies remain debated, encompassing neurological disorders (12), medial rectus muscle abnormalities (13), excessive near-work (10, 14), decompensated esophoria (15), and digital device overuse (5, 16).

A critical unresolved debate is whether uncorrected myopia during near work is an independent risk factor (3, 10, 17). While many patients routinely wear corrective lenses, their habits during prolonged near tasks are often overlooked. Furthermore, the established classification may not adequately reflect the spectrum of clinical presentations seen today.

Most myopic AACE patients retain some binocular function (7), suggesting a compensatory process might be involved in the pathogenesis. We hypothesize that the ability to maintain fusion at the far point (FP) without correction—a point requiring minimal accommodative convergence—could be a key differentiator, potentially separating patients into distinct pathogenic categories. As the FP is the natural viewing distance for uncorrected myopes and requires minimal accommodative effort, it provides a unique vantage point to assess vergence adaptation (18, 19). Assessing binocular function at this specific, ecologically relevant distance may be important for providing insights into the adaptive mechanisms underlying myopic AACE.

This study aims to investigate the pathogenesis of myopic AACE by introducing a novel classification based on fusion compensation ability at the FP and to examine its correlation with specific eye-use habits, particularly prolonged near gaze without myopia correction (MC).

## Materials and methods

This retrospective study adhered to the Declaration of Helsinki and was approved by the Ethics Committee of Wenzhou Medical University (2023-190-K-155-01). Informed consent was waived due to the anonymized retrospective data.

We enrolled 105 myopic AACE patients from the Affiliated Eye Hospital of Wenzhou Medical University (Jan 2020–Nov 2023). Inclusion criteria were: (1) myopia in both eyes; (2) AACE diagnosis; (3) complete records. Exclusion criteria included: ocular surgery, trauma, neurological disorders, or best-corrected visual acuity < 18/20 in either eye. A control group of 107 simple myopia patients was recruited.

All patients underwent detailed history-taking, including: routine MC habits, MC status during prolonged near gaze (e.g., smartphone use), daily near gaze duration, and time without MC during near tasks. Near gaze refers to sustained visual tasks at close distances, typically involving focused attention. The average distance was self-reported as <30 cm for myopes.

The refractive error for each participant was defined as the mean spherical equivalent of both eyes. Ocular deviation was measured with MC using prism and alternate cover test (PACT) and Maddox prism test (MPT) at distance (6 m) and near (33 cm). The deviation at the FP without MC was also measured. Binocular vision was assessed using Titmus, OPTEC3500, Worth 4-dot, and Bagolini striated lenses tests. The the convergence requirement at the FP (CRFP) was calculated as pupillary distance (mm) / FP (meters). The Bagolini test was chosen as the primary criterion for defining fusion at the FP because it assesses fusion under natural viewing conditions without artificial dissociation, closely simulating the state during uncorrected near work (20, 21). Conventional dissociative tests (e.g., Worth four-dot), while used complementarily at standard distances, are less suitable for evaluating the specific physiological condition at the FP (22).

AACE patients were divided into two groups based on the Bagolini test at the FP without MC: those maintaining fusion were assigned to the compensatory group; those who could not were assigned to the non-compensatory group. The FP was chosen as the diagnostic criterion due to its unique distance, allowing clear vision with minimal accommodative-convergence demand in uncorrected myopia. Fusion at the FP reflects compensation for esodeviation and thus functions as a classification marker.

Statistical analyses were performed using Graphpad Prism 9.4.1. Group comparisons used *t*-tests, Mann-Whitney U tests, chi-square tests, or Fisher’s exact tests. Correlations were assessed using Spearman’s rank coefficient. A *p*-value < 0.05 was significant.

## Results

### Baseline characteristics and eye-use habits

**FIGURE 1:**
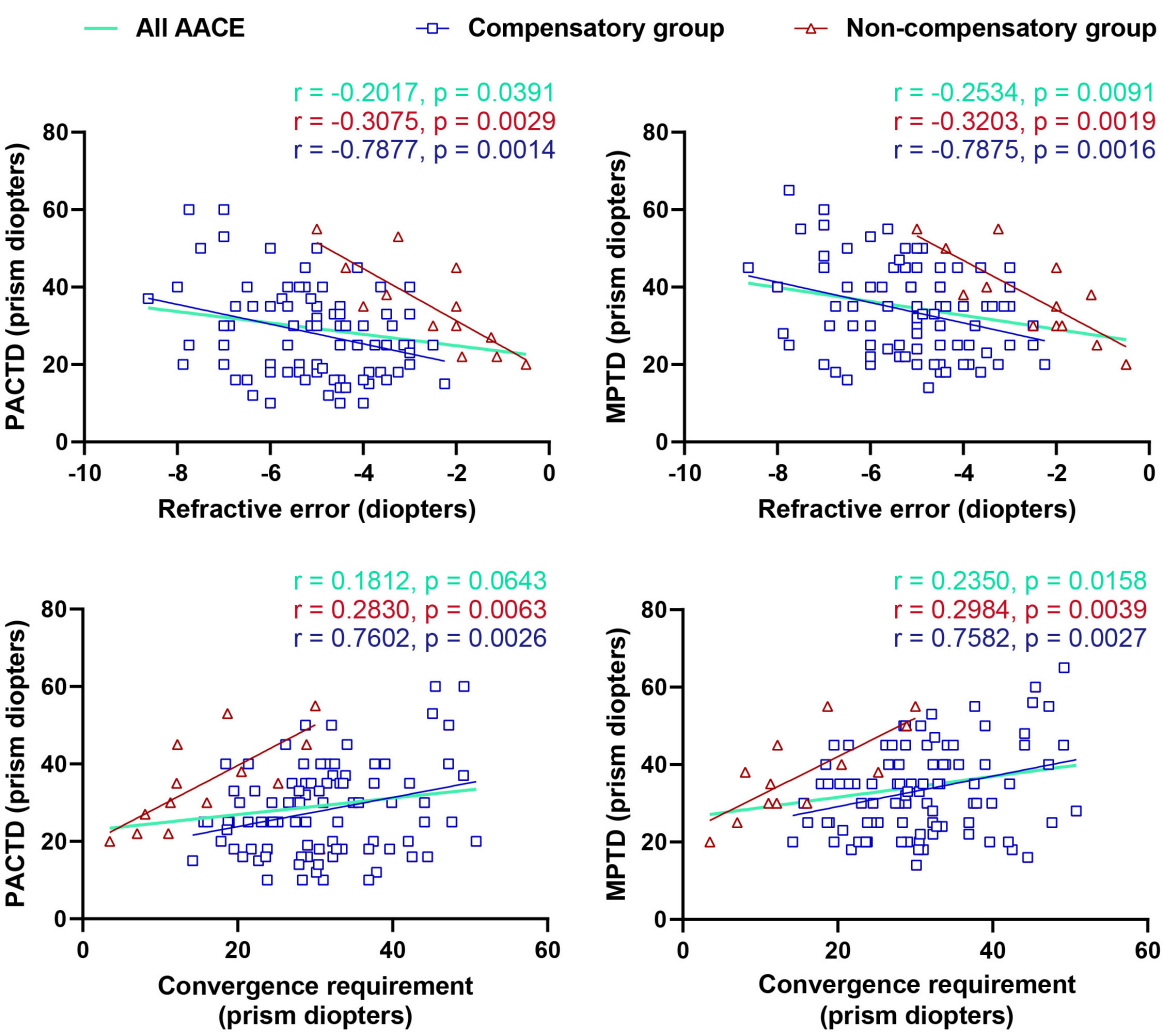
Correlation of refractive error and convergence requirement at far point (CRFP) with distance deviation (PACTD and MPTD) in all AACE patients, the compensatory group, and the non-compensatory group. AACE, acute acquired comitant esotropia; PACTD, prism and alternate cover test at distance; MPTD, Maddox prism test at distance.

The study included 105 AACE patients and 107 controls. The baseline characteristics were comparable between the two groups (Table 1). The median age of AACE patients was 26 years (interquartile range [IQR]: 18.5–33), while that of the controls was 25 years (IQR: 21–29), with no significant difference (*P* = 0.546). Regarding refractive error, the spherical equivalent was −4.74 ± 1.60 D in AACE patients and −4.70 ± 1.58 D in controls (*P* = 0.861). No significant difference was found in the rate of routine MC between groups (84.76% vs. 88.79% *P* = 0.387). However, the AACE group had a significantly higher rate (98.10% vs. 13.08%, *P* < 0.001) of near gaze without MC. Additionally, refractive error showed significant correlations with both PACTD and MPTD in the entire AACE cohort, as well as in both the compensatory and non-compensatory subgroups (all *P* < 0.05, Figure 1). Additionally, the AACE subgroups were compared with control group (Supplementary Tables 1, 2).

**TABLE 1:** Patients’ characteristics in AACE and control group.

Characteristics	AACE group (*n* = 105)	Control group (*n* = 107)	95% confidence interval	*P*-value
Age at visit (years)	26 (18.5, 33)	25 (21, 29)	1.00 (–1.00 to 3.00)^†⁣†^	0.546^a^
Age at AACE onset (years)	23 (17, 31.7)	—	—	—
Sex (M: F)	63: 42	55: 52	—	0.207^b^
Refractive error (diopters)	–4.74 ± 1.60	–4.70 ± 1.58	–0.04 (–0.47 to 0.39)^†^	0.861^c^
Rate of daily MC	89/105	95/107	—	0.387^b^
Rate of prolonged near gaze without MC*	103/105	14/107	—	<0.001^b^
Duration of daily near gaze time (hours)	6 (6, 8)	6 (4, 7)	1.00 (0.00 to 1.00)^†⁣†^	0.012^a^

Data are presented as median (interquartile range, 25th to 75th percentile) or mean ± standard deviation. AACE, acute acquired comitant esotropia; MC, myopia correction. *Daily MC does not include near-work.

^a^U test.

^b^Chi-square test.

*^c^*Un-paired *T*-test.

† Mean difference (95% confidence interval). ^†⁣†^Hodges-Lehmann estimator (95% confidence interval).

### Characteristics of the compensatory and non-compensatory groups

Based on the presence of fusion at the FP without MC, patients were categorized into compensatory (*n* = 92, 87.62%) and non-compensatory (*n* = 13, 12.38%) groups (Table 2).

**TABLE 2:** Patients’ characteristics in compensatory and non-compensatory groups.

Characteristics	All patients (*n* = 105)	Compensatory group (*n* = 92)	Non-compensatory group (*n* = 13)	95% confidence interval	*P*-value*
Age at AACE onset (years)	23 (17, 31.7)	25 (17, 34.45)	17 (14.5, 21)	6.10 (1.50 to 12.00)^†⁣†^	0.015^a^
Sex (M: F)	63: 42	55: 37	8: 5	—	0.904^b^
Rate of daily MC	89/105	84/92	5/13	—	<0.001^d^
Rate of prolonged near gaze without MC	103/105	90/92	13/13	—	>0.999^d^
Duration of daily near gaze time (hours)	6 (6, 8)	6 (6, 8)	6 (5.5, 8)	0.00 (–1.00 to 1.00)^†⁣†^	0.990^a^
Pupil distance (cm)	6.16 ± 0.31	6.16 ± 0.31	6.17 ± 0.37	–0.01 (–0.20 to 0.17)^†^	0.891^c^
Refractive error (diopters)	–4.74 ± 1.59	–5.04 ± 1.39	–2.57 ± 1.36	–2.48 (–3.29 to –1.66)^†^	<0.001^c^
Far point (cm)	21.62 (17.58, 27.13)	20 (16.67, 24.81)	50 (26.79, 66.67)	–23.95 (–33.33 to –10.77)^†⁣†^	<0.001^a^
**PACT (prism diopters)**					
Distance	30 (18.5, 36)	25 (18, 35)	35 (24.5, 45)	–7.00 (–15.00 to –0.00)^†⁣†^	0.040^a^
Near	20 (14, 30)	20 (12, 30)	35 (22.5, 42.5)	–12.00 (–20.00 to –5.00)^†⁣†^	0.003^a^
**MPT (prism diopters)**					
Distance	35 (25, 42.5)	33 (25, 40)	38 (30, 47.5)	–5.00 (–10.00 to 2.00)^†⁣†^	0.195^a^
Near	25 (18, 36)	25 (18, 35)	35 (30, 42.5)	–10.00 (–15.00 to –2.00)^†⁣†^	0.011^a^
PACT without MC at the FP	0 (0, 6)	0 (0, 4)	15 (14, 22.5)	–14.00 (–16.00 to –14.00)^†⁣†^	<0.001^a^
**Worth 4-dot^e^**					
Distance	16/105	16/92	0/13	—	0.210^d^
Near	43/105	43/92	0/13	—	0.001^d^
**Stereopsis (+): stereopsis (−)**					
Distance	15:90	15:77	0:13	—	0.206^d^
Near	51:54	51:41	0:13	—	<0.001^d^
CRFP (prism diopters)	29.01 (22.45, 34.53)	30.51 (24.44, 36.90)	12.20 (9.56, 22.84)	15.78 (10.13 to 20.46)	<0.001^a^

Data are presented as median (interquartile range, 25th to 75th percentile) or mean ± standard deviation. Comparing the compensatory and non- compensatory group. AACE, acute acquired comitant esotropia; PACT, prism and alternate cover test; MPT, deviation by Maddox prism test; FP, far point; MC, myopia correction.

^a^U test.

^b^Chi-square test.

^c^Un-paired *T*-test.

^d^Fisher’s exact test.

*^e^*The ratio of four dots. *P-values indicate comparisons between the compensatory and non-compensatory groups.

† Mean difference (95% confidence interval). ^†⁣†^Hodges-Lehmann estimator (95% confidence interval).

The compensatory group exhibited a consistent pattern wherein the MPT was significantly greater than the PACT at both distance and near fixation (*P* < 0.001), and both the MPT and PACT values were significantly larger at distance than at near (*P* < 0.001). Notably, the PACT measurement at the FP without MC was 0 PD (IQR: 0–4 PD). In contrast, the non-compensatory group demonstrated a different profile: MPT was not greater than PACT at distance and near fixation (*P* > 0.05), with no significant difference observed between distance and near deviations in both MPT and PACT (*P* > 0.05). Furthermore, this non-compensatory group showed a significantly larger PACT at the FP without MC, measuring 15 PD (IQR: 14–22.5 PD, Table 2).

Comparative analysis revealed that relative to the compensatory group, the non-compensatory group was characterized by a significantly younger onset age (median 17 vs. 25 years, *P* = 0.015), a lower daily rate of MC (*P* < 0.001), lower refractive error (mean −2.57 vs. −5.04 D, *P* < 0.001), larger angles of deviation (*P* < 0.05), worse near fusion (Worth 4-dot, *P* = 0.001), worse near stereopsis (*P* < 0.001), and a lower convergence requirement at the FP (CRFP, *P* < 0.001, Table 2).

The far point distance, measured objectively, was significantly closer in the compensatory group (median [IQR]: 20 [16.67, 24.81] cm) compared to the non-compensatory group (median [IQR]: 50 [26.79, 66.67] cm) (*P* < 0.001). This finding is consistent with the calculated CRFP values. Given that pupillary distance was similar between groups (*P* = 0.891), the markedly higher CRFP in the compensatory group directly results from their closer far point, indicating a substantially increased convergence requirement even for distant fusion.

### Relationship and comparison of CRFP with deviation measures

In the compensatory group, CRFP was positively correlated with both MPTD and PACTD (*P* < 0.05). CRFP was significantly greater than PACTD (*P* = 0.019), but not significantly different from MPTD (*P* = 0.054). In the non-compensatory group, CRFP was positively correlated with MPTD and PACTD (*P* < 0.001) but was significantly lower than both (*P* < 0.001, Figure 1).

## Discussion

Our study introduces a novel, clinically relevant classification for myopic AACE based on the presence of fusion compensation at the FP without MC. This approach successfully identified two distinct subgroups with significantly different clinical profiles, suggesting potentially divergent pathogenic mechanisms.

The overwhelmingly high rate of neglected MC during prolonged near work (98.10%) in AACE patients, contrasted with similar routine MC rates to controls, underscores that the critical risk factor is not myopia per se, but long-time near work without MC. This aligns with emerging literature on digital eye strain and AACE and offers a clear target for prevention: emphasizing consistent spectacle wear during all near tasks.

The compensatory group, representing the majority, is characterized by a larger deviation at distance relative to near, higher MPTD values relative to PACTD, and orthophoria at the FP without MC. The strong positive correlation between CRFP and deviation, and the near-equivalence of CRFP and MPTD, provides compelling evidence that the esodeviation in these patients may represent a maladaptive compensation for the excessive convergence demand incurred during uncorrected near gaze. Our direct measurement of the far point distance offers further support for this pathogenesis. The significantly closer far point in the compensatory group explains their elevated CRFP and indicates that their vergence system is maintained at a high tonic level, even under minimal accommodative demand at distance. This objective finding substantiates the concept of a system chronically adapted to excessive convergence. We postulate a mechanism: sustained voluntary convergence may overload the medial rectus muscles, potentially leading to adaptations such as contracture or hypertonia. The greater distance deviation may be attributed to insufficient relaxation of the tonically contracted medial recti muscles as convergence demand decreases. Nevertheless, patients’ retained fusional divergence ability may partially overcome this tone to maintain single binocular vision. Esotropia is thus revealed under dissociative conditions that break fusion. Future studies utilizing high-resolution orbital magnetic resonance imaging to quantify medial rectus muscle morphology, or employing electromyography to assess muscle activity, may be warranted to objectively test this hypothesis regarding muscle contracture and hypertonia.

Conversely, the non-compensatory group, with its younger age, lower myopia, larger angles, and absence of the classic deviation pattern, likely embodies a different pathophysiology. The inability to fuse even at the FP indicates a failure of compensatory mechanisms, possibly representing a decompensated esophoria (15) or a more profound disruption of vergence neural integration. This classification may help inform clinical management. It highlights the potential risk of deviation underestimation by conventional cover testing in compensatory patients, which might be attributed to fusional compensation. This suggests a potential need for supplemental tests (e.g., Maddox rod) to better determine the surgical target. These findings also lend support to the consideration of augmented surgical doses, as suggested in prior studies (23), which could potentially reduce the risk of under-correction and recurrence. Furthermore, the classification might offer guidance for postoperative strategies, such as considering binocular training for compensatory patients and alternative rehabilitation approaches for non-compensatory patients, thereby promoting a more personalized therapeutic framework.

Our proposed classification system can be contextualized within the historical framework of AACE subtypes. The non-compensatory group appears to share several characteristics with the acute-onset, fusion-disruptive types in the classic Burian-Miller classification (Types I and II). Similarly, the compensatory group may partially correspond to the traditional Type III (Bielschowsky), which has been associated with uncorrected myopia (1). However, our framework aims to build upon these classical descriptions by introducing a mechanism-based dichotomy centered on convergence adaptation at the far point. A key distinction lies in our focus on the ability to maintain fusion at this specific distance—a physiological marker not central to the classical system. This perspective may provide a more nuanced explanation for the clinical profile of a substantial proportion of contemporary AACE patients and could offer a basis for future subtyping research across the broader AACE spectrum.

This binary classification addresses limitations of the traditional Burian system. It provides a physiological basis for subtyping modern AACE cases and provides a potential explanation for clinical variations that confuse the existing schema. For example, the non-compensatory group’s features do not neatly fit into any classic type. From a clinical standpoint, this classification is pragmatic. Assessing fusion at the FP is simple and informs prognosis (compensatory likely better) and surgical planning, potentially explaining why surgery based on PACT measurements sometimes requires augmentation (23).

This study has limitations primarily stemming from its retrospective design and data collection methods. The reliance on patient-reported questionnaires for eye-use habits introduces the potential for recall bias. Furthermore, the retrospective nature limits causal inference and control for confounders. Future prospective studies incorporating objective measures of near-work behavior (e.g., using wearable sensors) are essential to validate our proposed classification and confirm these associations.

## Conclusion

Prolonged near gaze without myopia correction is a paramount risk factor for AACE. Classifying myopic AACE patients based on their fusion compensation ability at the far point reveals two distinct subtypes with different clinical characteristics. The compensatory type appears to be an abnormal adaptation to excessive convergence demand in myopia without MC during near tasks, while the non-compensatory type suggests a decompensated mechanism. This new classification system not only enhances our understanding of the pathogenesis but also provides a practical framework for clinical assessment and management.

## Data Availability

The raw data supporting the conclusions of this article will be made available by the authors, without undue reservation.
