# Supplementary material for: Compensatory versus non-compensatory types in myopic acute acquired comitant esotropia: a new classification based on fusion ability at the far point
Source: Front Med (Lausanne). 2026 Feb 23;13:1753378. doi: 10.3389/fmed.2026.1753378 (PMC12968022; doi:10.3389/fmed.2026.1753378)
Supplement: Supplementary file 1 [file Supplementary_file_1.docx]

Supplementary Table1. Patients’ characteristics in AACE compensatory groups and control group.

|  | Compensatory group  (n=92) | Control group  (n=107) | 95% confidence interval | P-value |
| --- | --- | --- | --- | --- |
| Age at visit (years) | 27 (20, 35.25) | 25 (21, 29) | 21.00 (19.00 to 23.00) ^††^ | 0.143^a^ |
| Age at AACE onset (years) | 25 (17, 34.45) | — | — | — |
| Sex (M: F) | 55:37 | 55: 52 | — | 0.236^b^ |
| Refractive error (diopters) | -5.04±1.39 | -4.70±1.58 | -0.34 (-0.76 to 0.07) ^†^ | 0.106^c^ |
| Rate of daily MC | 84/92 | 95/107 | — | 0.551^b^ |
| Rate of prolonged near gaze without MC | 90/92 | 14/107 | — | <0.001^b^ |
| Duration of daily near gaze time (hours) | 6 (6, 8) | 6 (4, 7) | 1.00 (0.00 to 1.00) ^††^ | 0.015^a^ |
| Data are presented as median (interquartile range, 25th to 75th percentile) or mean ± standard deviation.  ^a^ U test; ^b^ Chi-square test; ^c^ Un-paired T test.  ^†^Mean difference (95% confidence interval); ^††^Hodges-Lehmann estimator (95% confidence interval).  AACE, acute acquired comitant esotropia; MC, myopia correction. | | | | |

Supplementary Table2. Patients’ characteristics in AACE non-compensatory groups and control group.

|  | Non-compensatory group  (n=13) | Control group  (n=107) | 95% confidence interval | P-value |
| --- | --- | --- | --- | --- |
| Age at visit (years) | 26 (18.5, 33) | 25 (21, 29) | 13.00 (11.00 to 15.00) ^††^ | 0.005^a^ |
| Age at AACE onset (years) | 17 (14.5, 21) | — | — | — |
| Sex (M: F) | 8:5 | 55: 52 | — | 0.499^b^ |
| Refractive error (diopters) | -2.57±1.36 | -4.70±1.58 | 2.13 (1.23 to 3.04) ^†^ | <0.001^c^ |
| Rate of daily MC | 5/13 | 95/107 | — | <0.001^b^ |
| Rate of prolonged near gaze without MC | 13/13 | 14/107 | — | <0.001^b^ |
| Duration of daily near gaze time (hours) | 6 (5.5, 8) | 6 (4, 7) | 1.00 (-1.00 to 2.00) ^††^ | 0.257^a^ |
| Data are presented as median (interquartile range, 25th to 75th percentile) or mean ± standard deviation.  ^a^ U test; ^b^ Chi-square test; ^c^ Un-paired T test.  ^†^Mean difference (95% confidence interval); ^††^Hodges-Lehmann estimator (95% confidence interval).  AACE, acute acquired comitant esotropia; MC, myopia correction. | | | | |
